# Microarray Analysis of Gene Expression Profiles of *Schistosoma japonicum* Derived from Less-Susceptible Host Water Buffalo and Susceptible Host Goat

**DOI:** 10.1371/journal.pone.0070367

**Published:** 2013-08-05

**Authors:** Jianmei Yang, Yang Hong, Chunxiu Yuan, Zhiqiang Fu, Yaojun Shi, Min Zhang, Liuhong Shen, Yanhui Han, Chuangang Zhu, Hao Li, Ke Lu, Jinming Liu, Xingang Feng, Jiaojiao Lin

**Affiliations:** 1 Shanghai Veterinary Research Institute, Chinese Academy of Agricultural Sciences, Key Laboratory of Animal Parasitology, Ministry of Agriculture, Shanghai, People's Republic of China; 2 College of Veterinary Medicine, Sichuan Agricultural University, Ya'an, People's Republic of China; Queensland Institute of Medical Research, Australia

## Abstract

**Background:**

Water buffalo and goats are natural hosts for *S. japonicum* in endemic areas of China. The susceptibility of these two hosts to schistosome infection is different, as water buffalo are less conducive to *S. japonicum* growth and development. To identify genes that may affect schistosome development and survival, we compared gene expression profiles of schistosomes derived from these two natural hosts using high-throughput microarray technology.

**Results:**

The worm recovery rate was lower and the length and width of worms from water buffalo were smaller compared to those from goats following *S. japonicum* infection for 7 weeks. Besides obvious morphological difference between the schistosomes derived from the two hosts, differences were also observed by scanning and transmission electron microscopy. Microarray analysis showed differentially expressed gene patterns for parasites from the two hosts, which revealed that genes related to lipid and nucleotide metabolism, as well as protein folding, sorting, and degradation were upregulated, while others associated with signal transduction, endocrine function, development, immune function, endocytosis, and amino acid/carbohydrate/glycan metabolism were downregulated in schistosomes from water buffalo. KEGG pathway analysis deduced that the differentially expressed genes mainly involved lipid metabolism, the MAPK and ErbB signaling pathways, progesterone-mediated oocyte maturation, dorso-ventral axis formation, reproduction, and endocytosis, etc.

**Conclusion:**

The microarray gene analysis in schistosomes derived from water buffalo and goats provide a useful platform to disclose differences determining *S. japonicum* host compatibility to better understand the interplay between natural hosts and parasites, and identify schistosome target genes associated with susceptibility to screen vaccine candidates.

## Introduction

Schistosomiasis japonica is caused by the trematode *Schistosoma japonicum* and is one of the most prevalent zoonotic diseases in many Asian countries. In China, there are currently 365,770 human schistosomiasis cases, thus *S. japonicum* remains an important public health concern [1], [2]. Despite more than a half century of control efforts, there is no effective means for the control of this disease and no substantial progress in *S. japonicum* vaccine development has been made, thus treatment is mainly dependent on the drug praziquantel to kill the adult worm within the host. There is a wide range of hosts for *S. japonicum* that include at least 46 mammalian species, including humans and a variety of domestic and wild animals, such as rats, rabbits, dogs, cats, horses, yellow cattle, goats, sheep, donkeys, and monkeys, among others [3]. Previous studies have revealed that susceptibility of different types of hosts is varied, as mice, goats, and yellow cattle are more sensitive than rats and water buffalo for *S. japonicum* (Chinese strain) infections [1].

In China, uncontrolled schistosomiasis endemic areas are mostly distributed in marshland/lake and mountainous regions [4]–[6] and epidemiological surveys have revealed that domestic animals play important roles in schistosomiasis transmission [7]. Water buffalo and goats are major domestic animals reared in endemic areas of China, especially water buffalo can spread more eggs into the environment than humans or other animal hosts and, thus they are considered as primary transmission sources of schistosomiasis in endemic areas [8]–[9]. He et al. [10] infected mice, rats, guinea pigs, rabbits, goats, sheep, pigs, water buffalo, yellow cattle, horses and 12 other kinds of animals with *S. japonicum* under the same conditions and observed the development of parasites in these hosts for up to 60 weeks. Their results showed that the developmental rate of *S. japonicum* in these hosts was quite different, with the highest infection rate of 60.3% in goats, 43.6% in yellow cattle, and 1% in water buffalo and horses [10]. Water buffalo and goats act as major natural reservoir hosts for schistosomiasis in China although their susceptibility to schistosome infection is quite different, as goats are more susceptible to *S. japonicum* and result in higher rates of oviposition and sustain more severe pathological damage than water buffalo; however, the molecular basis of these differences remains unknown.

Several large-scale microarray analyses were recently performed using schistosomes to study gender-, stage-, and strain-specific gene expression of *S. japonicum* and *Schistosoma mansoni* [11]–[15]. The results of these studies suggested that gene and protein expression analysis in worms from different susceptible hosts can provide useful information to further elucidate the schistosome/host relationship. Recent studies in our laboratory revealed that schistosomes from a susceptible host (BALB/c mice), a less susceptible host (Wistar rat), and a non-permissive host (*Microtus fortis*, the reed vole) displayed different mRNA and protein expression profiles, and the gene expression analysis suggested that these three hosts may have different response mechanisms to schistosome infection [16]–[18]. These studies also indicated that the gene or protein expression profiles of schistosomes from natural hosts were different than those from laboratory animals. To better elucidate the susceptibility mechanism of schistosome in natural hosts and to identify molecules which might affect schistosome development, we infected water buffalo and goats with *S. japonicum* then analyzed and compared differences in gene expression profiles of the parasites obtained from the two hosts using microarray analysis. Our results will aid in screening vaccine candidates or new drug targets for the control of schistosomiasis in natural hosts dwelling within endemic areas.

## Materials and Methods

### Ethics Statement

All procedures were carried out in accordance with guidelines of the Association for Assessment and Accreditation of Laboratory Animal Care International (AAALAC). The animal study protocol was approved by the Animal Care and Use Committee of the Shanghai Veterinary Research Institute, Chinese Academy of Agricultural Sciences, People's Republic of China.

### Animals and infection

Male water buffalo and goats (n = 3 each), 15–18 months old, free of parasitic helminths and other infectious diseases were used for experimental infection. All animals were purchased from schistosome non-endemic areas with similar body weights for each host and were housed in covered pens, cared for by trained animal keepers, and fed hay and a commercial pelleted ration. *S. japonicum* (Chinese strain) cercariae were obtained from the snail maintenance room at Shanghai Veterinary Research Institute, Chinese Academy of Agricultural Sciences (Shanghai, China). Water buffalo were challenged percutaneously with 2000 cercariae of *S. japonicum* through the upper back using the cover glass method and goats were challenged percutaneously with 400 cercariae through the inguinal groove [19]. The cercariae were shed at 20–25°C before challenge to ensure maximum vitality.

### Sample collection and worm observation

The animals were sacrificed 7 weeks postinfection and the parasites were perfused through the hepatic portal vein. All worms were detached manually, counted, and the length and width of each was measured by the same investigator. The worms were measured using a motorized microscope equipped with auto camera ACT-2U (Nikon, Japan) and controlled by a Nikon image analysis software (NIS-Elements). Next, the worms were washed in phosphate-buffered saline (PBS) and then fixed in 2.5% glutaraldehyde phosphate buffer solution. Some of the worm samples were washed with PBS (pH 7.4) three times for 15–30 min each, fixed for 1.5 h with 1% osmic acid, washed three times as before, dehydrated with gradient alcohol (30%, 50%, 70%, 80%, 90%, 95%, and 100%), vacuum dried, spurted for 3 min using an ion sputtering instrument, and then observed via scanning electron microscopy (SEM) (JSM-6380LV; JEOL Ltd., Tokyo, Japan). The other samples were further dehydrated with acetone twice, embedded in an embedding medium, cut in ultrathin 70-nm sections and then observed via transmission electron microscopy (TEM) (Hitachi H-600; Hitachi Medical Corporation, Tokyo, Japan).

### RNA extraction and microarray analysis

Worm samples were collected and stored in RNAlater RNA stabilization reagent (Ambion, Carlsbad, CA, USA). Total RNA was extracted from the parasites(10 pairs each animal) collected from water buffalo and goats using Trizol reagent (Invitrogen Life Technologies, Carlsbad, CA, USA) and purified using the RNAeasy mini kit (Qiagen GmbH, Hilden, Germany) according to the manufacturer's instructions. The RNA integrity and quality was evaluated using the Agilent 2100 bioanalyzer (Agilent Technologies, Inc., Santa Clara, CA, USA).

The microarrays used to analyze the gene expression profiles in schistosomes from water buffalo and goat were constructed by Agilent Technologies, Inc. and included 13,821 contiguous sequences (contigs) plus proprietary positive and negative controls. Contigs were based on the nucleotide sequences from a recent *S. japonicum* database. Full details of this schistosome microarray design have been deposited in the Gene Expression Omnibus (GEO) database with the platform accession number GPL10987. Microarrays were printed in an 8×15 k feature format.

A 200-ng aliquot of total RNA from each sample was converted into complementary RNA, labeled with the fluorophore cyanine 3-CTP (CY3c) and hybridized according to the manufacturer's instructions (Agilent Technologies, Inc.; One-Color Microarray-Based Gene Expression Analysis). Samples were examined at wavelengths of A260 and A550 using the ND-1000 spectrophotometer (Thermo Fisher Scientific, Waltham, MA, USA) to determine yield, concentration, amplification efficiency, and abundance of CY3c. Two technical replicates were performed for each sample and three independent biological replicates were designed for each host.

### Feature extraction and data analysis

Microarrays were scanned using an Agilent Microarray Scanner (G2565BA) at a wavelength of 550 nm. Hybridized slides were scanned as tagged image files (TIFF) and processed with the Feature Extraction 9.5.3.1 Image Analysis program (Agilent Technologies, Inc.) to produce standardized data for statistical analysis. All slides were assessed for background evenness by viewing the TIFF image using Feature Extraction. Feature extracted data was analyzed using the GeneSpring GX statistical tool (version 7.3.1; Agilent Technologies, Inc.). Microarray data were normalized using a normalization scenario for ‘Agilent FE one-color,’ which included ‘Data Transformation: Set measurements less than 5.0 to 5.0’, ‘Per Chip: Normalize to 50th percentile’, and ‘Per Gene: Normalize to median’.

Data sets were further analyzed based on one-color experiments that have been published elsewhere [20]. The gProcessed Signal values were determined by the GeneSpring GX statistical tool using Feature Extraction software (Agilent, Inc.), including aspects of signal/noise ratio, spot morphology, and homogeneity. The gProcessed Signal represents signals after localized background subtraction and includes corrections for surface trends. Features were deemed *absent* when the processed signal intensity was >2-fold of the value of the processed signal error value. Features were deemed *marginal* when the measured intensity was at a saturated value or if there was a substantial amount of variation in the signal intensity within the pixels of a particular feature. Features that were neither *absent* nor *marginal* were deemed *present*. Data points were included only if they were *present* or *present/absent* and probes or contigs were retained if all data points were *present* or *present/absent*. All microarray data were submitted to the Gene Expression Omnibus public database under the accession number GSE24615. All statistical analyses were performed using R statistical language software (www.r-project.org/) and the *q*-value was estimated using the false discovery rate (FDR) as a control [21]–[22]. Heatmap and principal component analysis (PCA) were plotted using Java Treeview software (Stanford University, Stanford, CA, USA) and a multidimensional scaling algorithm [23].

### Gene ontology and pathway pattern analysis

Further analysis was performed using Blast2Go Batch BlastX software (6-frame translation protein homology; http://www.blast2go.de) [24] to evaluate differences in annotation between two groups of data. The analysis of gene ontology (GO) terms associated to genes considered differentially expressed in Group B (schistosomes from water buffalo) compared to Group G (schistosomes from goats) was performed using the combined graph function of the software. GO correlations with relative gene expression values were made using ErmineJ software [25]. Kyoto Encyclopedia of Genes and Genomes pathway patterns of differentially expressed genes of interest were analyzed using the SBC Analysis System (http://sas.ebioservice.com/).

### Real-time polymerase chain reaction (PCR) validation

Real-time PCR was used to validate a subset of genes predicted to be differentially expressed in the microarray experiment. All gene-specific primers were designed using PRIMER3 software (http://frodo.wi.mit.edu/primer3/input.htm). Purified RNA from mixed parasite samples from each animal in each group was used for reverse transcription in a final volume of 20 µL using the PrimerScript RT kit with gDNA Eraser (Cat# DRR047; Takara Bio, Inc., Shiga, Japan). Products were amplified using the SYBR Premix Ex Taq (Cat#DRR041A; Takara Bio, Inc.) in an ABI 7500 Real-time System (Applied Biosystems) with the following profile: 50°C for 2 min, 95°C for 30 s; 40 cycles of 95°C for 5 s and 60°C for 34 s; 95°C for 15 s, and 60°C for 1 min. Each reaction was performed using 20 µL of cDNA from the RT reaction in a final volume of 50 µL. Expression levels of *S. japonicum* nicotinamide adenine dinucleotide dehydrogenase (GenBank accession no.: AY812950) were used as endogenous controls within each sample. Relative levels of gene expression were calculated using the 2^−ΔΔCT^ method [26]. The correlation of microarray and qPCR analysis was performed by SPSS 16.0 [27].

## Results and Discussion

### Morphology of schistosomes derived from two natural hosts

**Figure 1:**
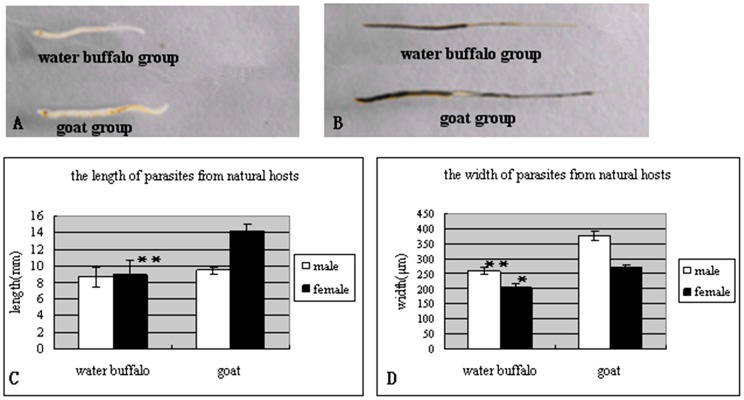
Comparison of the length and width of worms from water buffalo and goats at week 7 postinfection. (A) male worms; (B) female worms; (C) parasite length; (D) parasite width. *, *p*<0.05; **, *p*<0.01. The number of worms compared per group was at least n = 50 for females and males, respectively, and included worms from all animals.

The infected goats displayed more serious disease manifestations than the water buffalo, including diarrhea, athrepsia, egg-deposition, and pathological liver damage [28]. The worm recovery rate in water buffalo was 2.9±1.05%, which was much lower than that in goats (49.50±5.50%). Compared to schistosomes from a third natural host, yellow cattle, schistosomes from water buffalo presented growth and paring retardation [29]. In this study, schistosomes from water buffalo also showed growth retardation compared to parasites from goats (Figure 1A and B). Parasitic length and width from the two hosts were obviously different, as results showed that the female/male worm lengths from water buffalo and goats were 8.86±1.86 mm/8.66±1.23 mm and 14.20±0.84 mm/9.40±0.55 mm, respectively, and the width of female/male worms from water buffalo and goats were 202.98±12.33 µm/259.54±14.57 µm and 267.40±15.24 µm/377.05±15.97 µm, respectively (Figure 1C and D).

**Figure 2:**
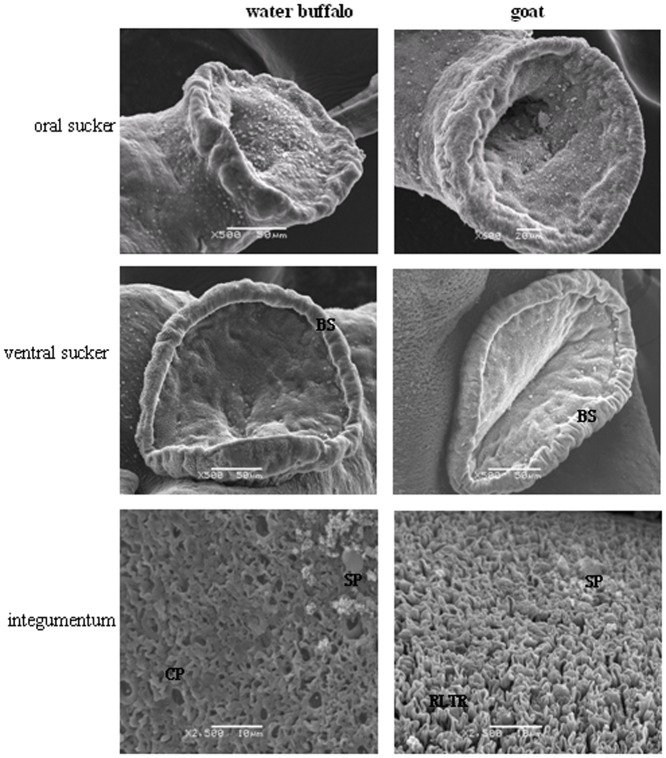
Comparisons of the oral suckers, ventral suckers, and the integument of male worms derived from water buffalo and goats by SEM. BS, border spine; CP, cortical pore; RLTR, rope-like tegumental ridge; SP, sensory papillae.

**Figure 3:**
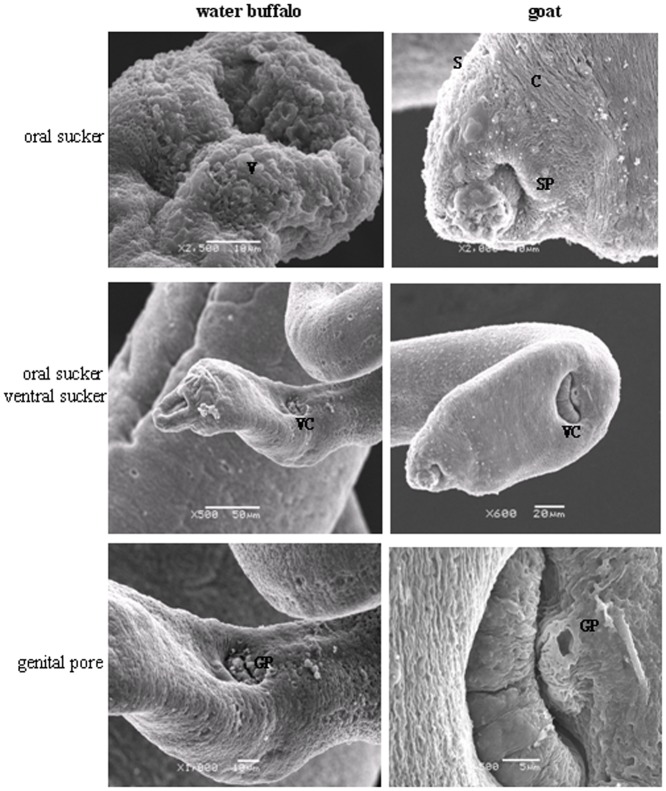
Comparisons of the oral suckers, ventral suckers, and the genital pores of female worms derived from water buffalo and goats by SEM. V, vacuole; C, crest; S, spine; SP, sensory papillae; VC, ventral sucker; GP, genital pore.

Internal structure analysis at the ultrastructural level by SEM and TEM showed differences between worms from the two natural hosts. Of male schistosomes from water buffalo, the oral and ventral suckers were crimpled, tension loosed, and the esthema mastoid processes in the integument were flattened and vacuolated, while the surface crest and sensory papillae in male worms from goats were affluent, compact, and well arranged (Figure 2). The female schistosomes from water buffalo had collapsed oral suckers on both sides, depressed ventral suckers, and poorly distinguished genital pores beneath the ventral suckers, while female worms from goats were affluent with more esthema mastoid processes in the oral suckers and obvious genital pores next to ventral suckers (Figure 3).

**Figure 4:**
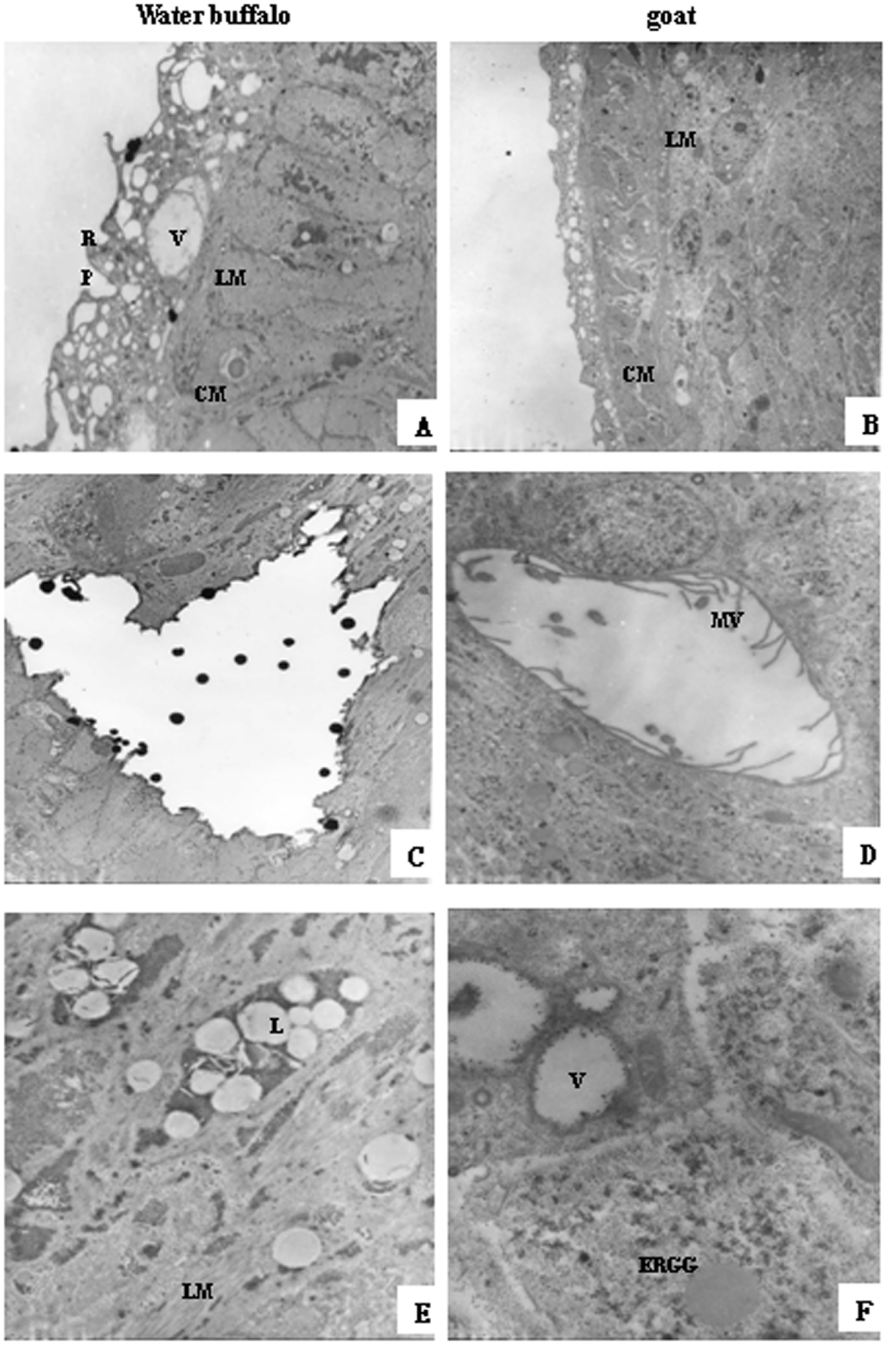
Comparisons of male worms derived from water buffalo and goats by transmission electron microscopy (TEM). (A, B) tegument, 2,000× magnification; (C–F) subtegument and inner structures; (C, D) 3,000× magnification; (E, F) 10,000× magnification. R, ridge; P, pore; V, vacuoles; LM, longitudinal muscle; CM, outer circular muscle; MV, microvilli; L, lipid droplet; ERGG, endoplasmic reticulum glycogen granule.

**Figure 5:**
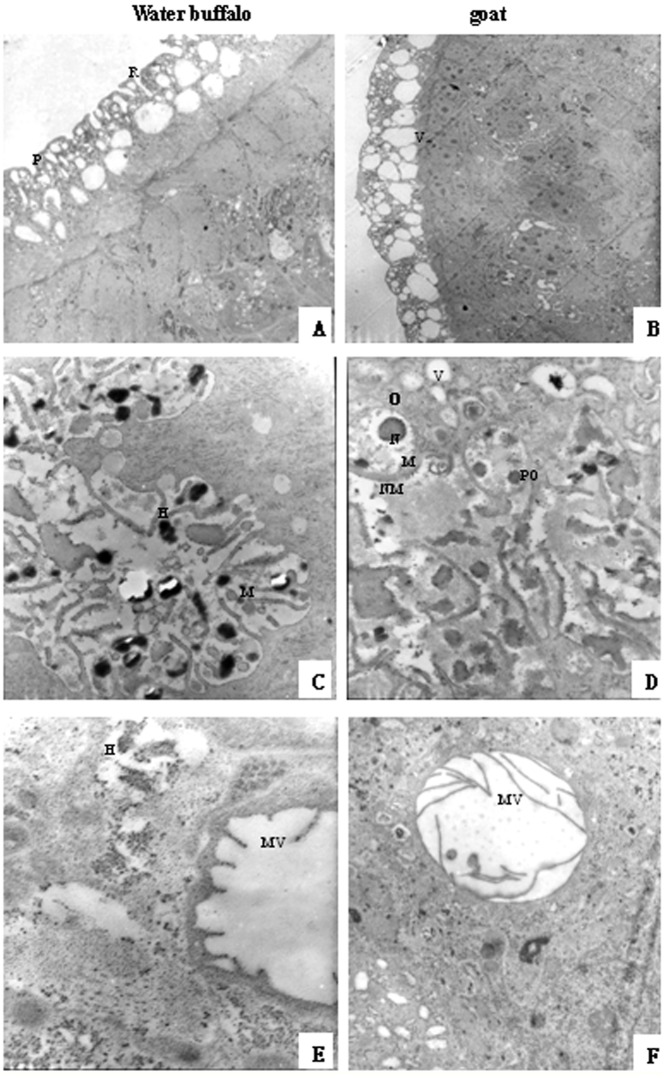
Comparisons of the female worms derived from water buffalo and goats by TEM. (A, B) tegument, 2000× magnification; (C–F) subtegument and inner structure, 12,000× magnification. H, heterochromatin; M, mitochondria; O, oogonia; N, nuclei; NM, nuclear membrane; PO, primary oocytes.

A comparison of male worms from water buffalo and goats using TEM showed that those from water buffalo had more vacuolar structures in the teguments, no internal microvilli, and the cytoplasmic organelles were dissolved (Figure 4). The female worms from water buffalo and goats had no obvious differences in the tegument, but there were more and longer internal microvilli in female worms from goats than from water buffalo (Figure 5).

### Global gene expression profiles in schistosomes from water buffalo and goat

**Figure 6:**
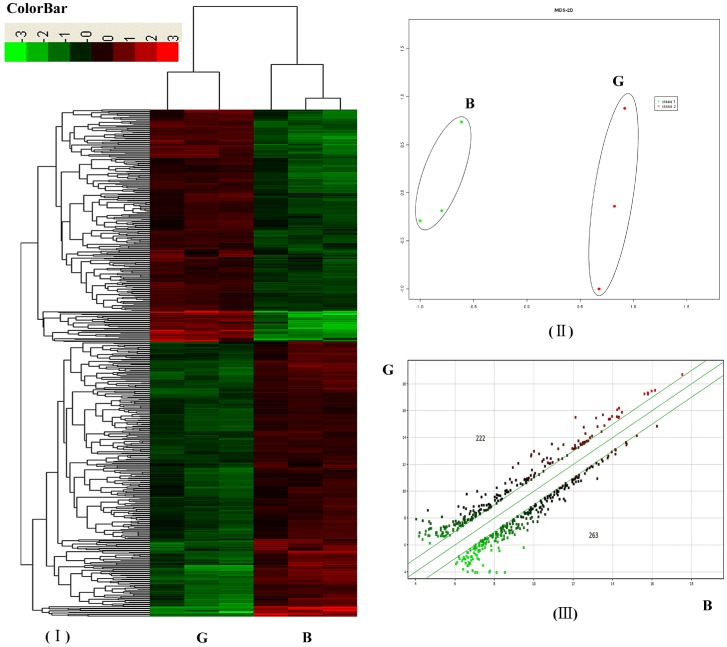
Transcription profile analysis of significantly differentiated expressed genes in *S. japonicum* from water buffalo (group B) and goats (group G). (A) Hierarchical clustering using differentially expressed genes (probe sets) (*p*<0.05; FC>2); (B) Principal component analysis (PCA) of transcript profiles from groups B and G; (C) A scatter plot comparing groups G and B. The number at the upper left denotes up-regulated genes and the number at the lower right denotes down-regulated genes (*p*<0.05; FC>2).

Three biological replicates of schistosomes from each host were evaluated and the correlation between biological replicates was 0.99 for each host. All data have been deposited in the Gene Expression Omnibus database maintained by the National Center for Biotechnology Information (GEO Series accession no.: GSE24615) (http://www.ncbi.nlm.nih.gov/geo/). We used 485 significant transcript expression values of schistosomes from the two natural hosts (*p*<0.05, fold change (FC)>2) for hierarchical clustering. Different profiles could be clearly identified among schistosomes from water buffalo (Group B) and goats (Group G). Two main clusters separated the schistosome genes among the two different hosts and the gene expression patterns of groups B and G were clustered (Figure 6I). To evaluate the overall data structure, we plotted the first two principal components of a PCA to capture the overall variance of the samples in two dimensions. This analysis clearly separated the data into two subgroups, which clustered the biological replicates together and separated the samples by the host from which the schistosomes were derived (Figure 6II).

**Figure 7:**
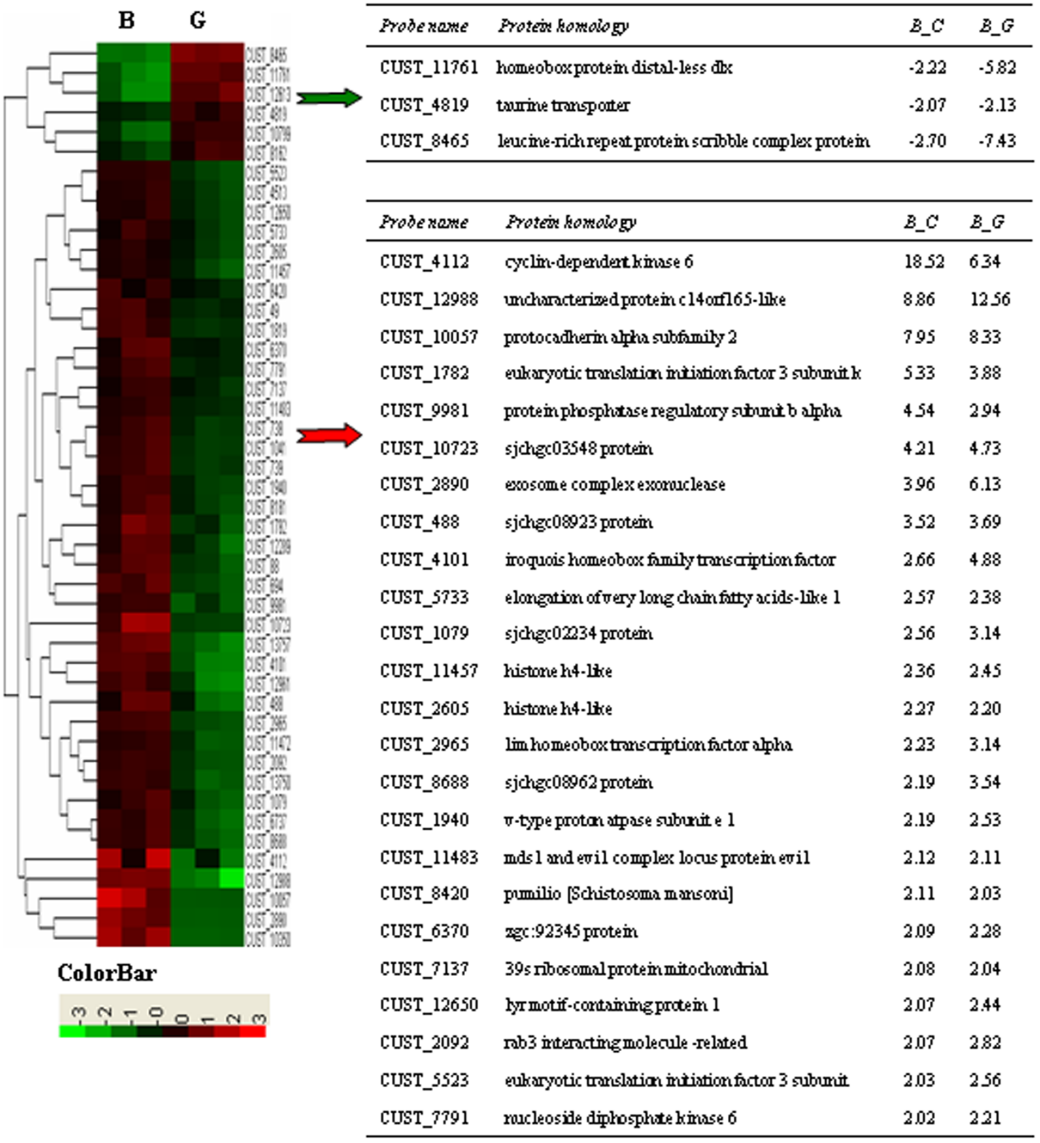
Heatmap clustering of common differentially expressed genes. The right tables listed the common differentially expressed genes (*p*<0.05; FC>2). Schistosomes from water buffalo, yellow cattle, and goats were simplified as groups B, C, and G respectively. “-” indicates downregulation. FC is represented as the mean value. The tables do not contain genes without protein homology descriptions.

For genes to be considered differentially expressed, a ≥2 FC in gene expression was required with a *t*-test *p*-value <0.05. Compared to parasites from water buffalo, the distribution of the up- and down-regulated genes are shown in the scatter plot in Figure 6-III(222 up-regulated and 263 down-regulated). More differentially expressed genes were found in schistosomes between water buffalo and goats than between water buffalo and yellow cattle (485 vs. 69) because the members of the latter pair were more closely related to each other phylogenetically. Most differentially expressed schistosome genes between water buffalo and yellow cattle are also found in schistosomes between water buffalo and goats (46/69 vs. 46/485). A heatmap is shown in Figure 7 and a gene list is shown in Table S1 and S2 for the common differentially expressed genes. The majority of them were analyzed in detail in our previous report [30]. In this study, we identified several additional important differentially expressed genes between worms from water buffalo and goats.

### Real-time PCR validation of microarray data

Subsets of genes with different expression levels and various biological functions in schistosomes from water buffalo and goats were selected for real-time PCR analysis to validate the microarray transcription results. The primer sequences and validation results are listed in Table 1. The real-time PCR results matched the microarray data very well, including the directionality and fold changes, a significant correlation of 0.817 was observed in our data (Spearman's Rho, p<0.01, n = 9), thereby validating the microarray results.

**Table 1:** Details of real-time RT-PCR primers and results of confirmation.

ProbeName	Primer(5′-3′)	Size (bp)	Real-time FC^a^	Microarry FC^b^	Description
CUST_551	FP:agatcgcgttcaaacaacaaRP:atctgccggatatgaaccaa	196	−2.63	−6.84	Expressed protein
CUST_12936	FP:gaacgtgatgctgttgttgcRP:atcctcgacatcccaatcag	192	−2.64	−7.27	Prostatic spermine-binding protein precursor (SBP)
CUST_4819	FP:ttaagcgggatcaatggaagRP:caccacgacgtgttaattgc	210	−2.05	−2.14	Solute carrier family 6 (ko:K05045)
CUST_12988	FP:ctgctgtggagggaatgtttRP:tggaggattccaggtttcag	219	50	12.56	Putative uncharacterized protein C14orf165
CUST_4112	FR:gttattggatttcccgctcaPR:atggcaatgaaagtgcatca	199	3.34	6.34	Cyclin-dependent kinase 6, CDK6
CUST_10350	FR:ggattgattccgccattacPR:gaatggcagtattggttgacg	198	4.75	6.88	Asparagine-rich protein (Ag319) (ARP)
CUST_10723	FR:tgtgccgttattgcgtttagPR:attatcgcttttgccgtcag	178	2.11	4.73	Expressed protein
CUST_4101	FR:atactggtgagcggcctatgPR:gcattcgcaaaccatgtaga	230	3.52	4.88	Iroquois homeobox protein 3, IRX-3
CUST_5523	FR:gcgttcgccaattgaattatPR:tgttgtattgggtggggatt	184	5.2	2.56	Putative eukaryotic translation initiation factor 3 subunit (eIF-3)

a Fold change (FC) is the ratio of gene expression in schistosomes from water buffalo compared to those from goats; *p*<0.05, FDR<0.1;

b Mean FC in real-time PCR results for validation.

### GO functional distribution and pathway pattern analysis of differentially expressed genes in schistosomes from water buffalo and goats

**Figure 8:**
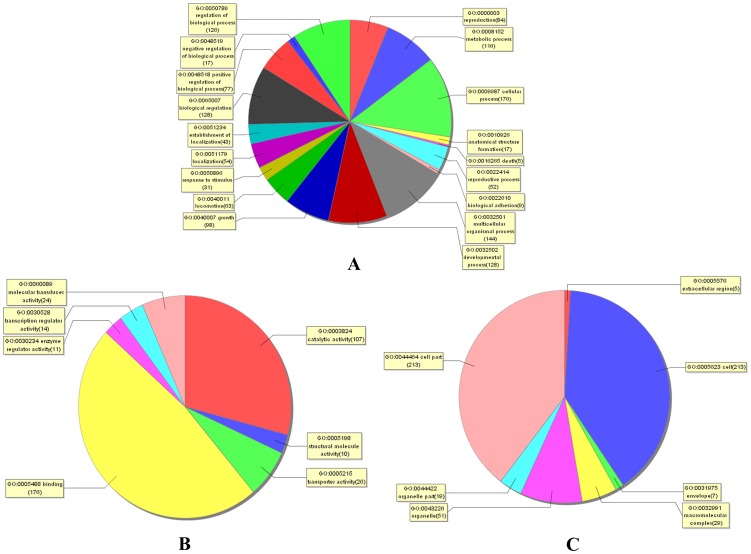
Distribution of gene ontology (GO) terms for the differentially expressed genes in schistosomes from water buffalo and goats. A pie plot showing GO classifications of the biological processes (A), the molecular functions (B), and the cellular components (C). The graph excluded data without assigned GO terms.

The differentially expressed genes were mainly involved in biological regulation, developmental processes, growth, metabolic processes, cellular processes, and reproduction, (Figure 8A). Molecular functional analysis revealed that most of these molecules were involved in binding, catalytic activity, transportation, molecular transduction, transcription regulation, and enzymatic regulation (Figure 8B). Cellular component analysis showed that most of these molecules were components of the cell, cell parts, organelles, organelle parts, and envelope (Figure 8C). GO analysis and other bioinformatic techniques were further applied to predict/analyze possible functions of the identified bioactive molecules.

### Up-regulated genes in schistosomes from water buffalo compared with those from goats

Compared with schistosomes from goats, many genes related to lipid metabolism (prostaglandin synthase 1(PGS-1), acyltransferase-like family member 8 (ACL-8), acetylcholinesterase 1 (AChE-1), and degenerative spermatocyte homolog 1(DEGS-1)), genetic information processing (DNA primase, nucleoside diphosphate kinase (NDK), trans-inducing factor, etc.), folding, sorting and degradation (26S proteasome, bromodomain and tryptophan-aspartic acid (WD) repeat domain containing 3 (BRWD3), and ribosomal RNA-processing protein 4 (RrP4)) were significantly up-regulated in schistosomes from the less-susceptible host water buffalo (Table 2).

**Table 2:** Selected genes overexpressed in schistosomes from water buffalo compared to those from goats.^a^

Pathway	Probe name	Accession	Gene and/or protein homology	Fold change	*p* value	FDR
**Lipid Metabolism**	**Glycerophospholipid metabolism**					
	CUST_3764	CNUS0000098059	Prostaglandin synthase 1	2.19	0.021	0.007
	CUST_7726	CNUS0000102024	Acetylcholinesterase 1	2.71	0.036	0.007
	**Glycerophospholipid metabolism & Glycerolipid metabolism**					
	CUST_8880	CNUS0000103178	Acyltransferase-like family member 8	2.57	0.026	0.008
	**Sphingolipid metabolism**					
	CUST_6882	CNUS0000101178	Degenerative spermatocyte homolog 1	2.45	0.021	0.008
**genetic information processing**	**Purine metabolism, Pyrimidine metabolism**					
	CUST_3246	CNUS0000097541	DNA primase small subunit	2.02	0.023	0.01
	CUST_9849	CNUS0000104147	Nucleoside-diphosphate kinase	2.08	0.001	0
	**Transcription**					
	CUST_9094	CNUS0000103392	Transcription initiation factor TFIIE alpha	2.34	0.018	0.008
	**Spliceosome**					
	CUST_11072	CNUS0000105370	Hypothetical protein	2.62	0.036	0.01
	CUST_488	FN317168	SJFCE0697	3.69	0.022	0.008
	CUST_7382	CNUS0000101679	Expressed protein	4.53	0.019	0.007
	**Translation**					
	CUST_6052	CNUS0000100348	Small subunit ribosomal protein S27Ae	4.71	0.002	0.007
**Folding, Sorting and Degradation**	**Proteasome**					
	CUST_3409	CNUS0000097704	26S proteasome regulatory subunit N12	3.26	0.001	0.006
	**Ubiquitin mediated proteolysis**					
	CUST_9175	CNUS0000103473	Bromodomain and WD repeat domain containing 3	2.55	0.001	0.006
	**RNA degradation**					
	CUST_2890	CNUS0000097185	RNA-binding protein Rrp4 and related proteins	6.13	0.020	0.007

a This list includes the probe name, gene accession number, fold change, as well as *p*- and *q*-values for protein homology (BlastX). In the gene accession columns, data with identifiers such as CNUS0000098407 were retrieved from the *S. japonicum* genome project database (LSBI; http://lifecenter.sgst.cn/schistosoma/cn/genomeProject.do; in Japanese) and those with identifiers FN326902 were retrieved from The European Molecular Biology Laboratory database (http://www.ebi.ac.uk/ena/). The FDR (*q*-value) was determined using the false discovery rate as a control using R statistical language (http://www.bioconductor.org/packages/release/bioc/html/qvalue.html).

#### Lipid metabolism

The genes associated with lipid metabolism were overexpressed in schistosomes from water buffalo compared to those from goats, but the differentially expressed genes varied among the hosts. The up-regulated genes in schistosomes from water buffalo included PGS-1, ACL-8, AChE-1, and DEGS-1. Prostaglandins are found in most tissues and organs, and synthesized by almost all nucleated cells from essential fatty acids as autocrine and paracrine lipid mediators. Prostaglandins ligate G-protein-coupled receptors, a sub-family of cell surface seven-transmembrane receptors. Ten prostaglandin receptors on various cell types were reported recently and the diversity of the receptors indicates that prostaglandins act on an array of cells and have a wide variety of effects, such as inducing constriction or dilation in vascular smooth muscle cells, aggregation or disaggregation of platelets, and controlling hormone regulation, cell growth, and other processes [31]. Acylation and deacylation are the most common eukaryotic protein post-translational modifications. Acyltransferase functions as an acylation/deacylation catalytic protein and may have multiple functions in regulation of protein biological activity and gene expression.

The parasites derived from water buffalo were stunted and increased expression of nervous system- and transport-related genes when compared to those from yellow cattle [29]. In this study, the AChE-1 and DEGS-1 genes were up-regulated in worms from water buffalo compared to those from goats. AChE-1 is a serine protease that belongs to the carboxylesterase enzyme family and hydrolyzes the neurotransmitter acetylcholine at the synapses and can also hydrolyze butyrylthiocholine. AchE-1 is found mainly at neuromuscular junctions and cholinergic brain synapses where it serves to terminate synaptic transmission. Although AChE-1 is detected at all growth stages of *Caenorhabditis elegans*, it is more abundant in larval stages than in embryos or adults [32]. DEGS-1 encodes a membrane-bound fatty acid desaturase, which is responsible for forming double bonds into specific positions in fatty acid molecules. DEGS-1 can up-regulate cyclin D1 expression and activation of the transcription factor nuclear factor kappa-light-chain-enhancer of activated B cells [33]. Cyclin D1 plays a pivotal role in cell-cycle transition through the G1 phase and regulates cyclin-dependent kinase 6, which, in turn, regulates schistosome embryogenesis and worm paring through the transforming growth factor beta signaling pathway [34], [35]. DEGS1 overexpression inhibited biosynthesis of the epidermal growth factor receptor (EGFR) [36] and in this study, the EGFR gene was down-regulated (CUST_3717, FC −4.52) in schistosomes from water buffalo, which might be attributable to the DEGS1 over-expression.

#### Genetic information processing

Biological phenotypes were determined according to gene expression. The genetic information processing-associated genes showed significant differential expression in schistosomes among the hosts. Schistosomes cannot synthesize purines alone, thus they are dependent on a supply from the host [37]. Our results determined that purine and pyrimidine metabolism-associated genes were overexpressed in schistosomes from water buffalo compared to than those from goats, including DNA primase (CUST_3246) and nucleoside-diphosphate kinase (CUST_9849). In addition to its catalytic activity of phosphorylation, NDK reportedly regulates growth and development [38] and is considered a housekeeping enzyme for DNA and RNA synthesis. However, NDK, as well as other transcription-, splicesome-, and translation-associated genes were upregulated in the less susceptible host water buffalo, suggesting that they are important for parasite retardation (Table 2).

#### Folding, Sorting, and Degradation

The differentially expressed molecules found in this class included posttranslational modification molecules, as 26S proteasome (CUST_3409), BRWD3 (CUST_9175), and RrP4 (CUST_9175) were up-regulated in schistosomes from water buffalo. The 26S proteasome is at the executive end of the ubiquitin proteasome pathway (UPP) for the controlled degradation of intracellular proteins. In eukaryotes, the UPP is essential for proteostasis, in which misfolded or otherwise defective proteins as well as short-lived regulatory proteins are eliminated by degradation [39]. The UPP regulates many fundamental cellular processes, such as protein quality control, DNA repair, cell cycle regulation, antigen processing, and signal transduction [40]. Lysine acetylation is similar to protein phosphorylation in its prevalence in posttranslational modifications and also has a large effect on the physicochemical properties of the modified residues. Protein recruitment to macromolecular complexes by acetylated lysine residues is mediated by bromodomains (BRDs), which are evolutionarily highly conserved protein interaction modules that recognize ε-N-lysine acetylation motifs [41]. The WD repeat proteins, BRWD1 and BRWD3, also contain tandem BRDs. Members of this family are involved in a variety of cellular processes, including cell cycle progression, signal transduction, apoptosis, and gene regulation [42]; however, little is known regarding the biological function of BRWD3. In Drosophila, BRWD3 function has been genetically linked to the janus kinase- signal transducer and activator of transcription pathway [43]. Mutations in mice revealed a role for BRWD1 in spermiogenesis and oocyte–embryo transition [44]. RNA-binding proteins (RBPs) bind to either double-strand or single-strand RNA molecules through RNA recognition motifs. Thus, RBPs may regulate RNA translation and post-transcriptional events, such as RNA splicing and editing.

### Down-regulated genes in schistosomes from water buffalo compared with those from goats

Compared with schistosomes from goats, many genes related to signal transduction (i.e., Akt, ribosomal s6-p90 kinase (RSKN-1), mitogen-activated protein kinase kinase 1 (MEK-1), MEK-6, protein kinase-18 (KIN-18), EGFR, 70-kDa heat shock protein (HSP70), dishevelled (Dsh), etc.), endocrine function (Akt, RSKN-1, MEK-1), development (MEK-1, EGFR), reproduction (SBP), immune function (MEK-1), endocytosis (MEK-1, HSP70, EGFR, partitioning-defective 3 homolog (PAR-3), etc.), amino acid metabolism (aspartate aminotransferase, dopamine beta-hydroxylase, and aromatic-L-amino-acid decarboxylase), carbohydrate metabolism (PI3K-C2α, PI4Kα, pyruvate carboxylase, and galactokinase 2 (GALK2)), glycan biosynthesis, and metabolism (glycosylphosphatidylinositol anchor attachment protein 1 (GPAA1) and glycoprotein 2 (GLY-2)) were significantly down-regulated in schistosomes from the less-susceptible host water buffalo (Table 3).

**Table 3:** Selected downregulated genes in schistosomes from water buffalo compared with those from goats.^a^

Pathway	Probe name	Accession	Gene and/or protein homology	Fold change	*p* value	FDR
**Signal transtruction**	**MAPK signaling pathway**					
	CUST_11642	CNUS000010594	Akt1;RAC serine/threonine-protein kinase	−6.92	0.022	0.005
	CUST_12915	CNUS0000107219	Protein RSKN-1	−2.48	0.018	0.005
	CUST_3675	CNUS0000097970	MAPK/ERK kinase 1(MEK-1)	−4.15	0.0002	0
	CUST_9010	CNUS0000103308	MAPK/ERK kinase 6(MEK-6)	−2.91	0.003	0
	CUST_9047	CNUS0000103345	Protein KIN-18	−15.09	0.0003	0
	CUST_3717	CNUS0000098012	Epidermal growth factor receptor	−4.52	0.004	0.005
	CUST_9487	CNUS0000103785	Probable beta-arrestin	−2.33	0.040	0.007
	CUST_5301	CNUS0000099597	Heat shock 70 kDa protein cognate 4	−2.24	0.024	0.01
	**ErbB signaling pathway**					
	CUST_11642	CNUS0000105941	Akt1;RAC serine/threonine-protein kinase	−6.92	0.022	0.005
	CUST_3675	CNUS0000097970	MAPK/ERK kinase 1	−4.15	0.0002	0
	CUST_5301	CNUS0000099597	Heat shock 70 kDa protein cognate 4	−2.24	0.024	0.01
	**Wnt signaling pathway, Notch signaling pathway**					
	CUST_5485	CNUS0000099781	Dishevelled protein(Dsh)	−4.48	0.001	0.005
**Endocrine System**	**Progesterone-mediated oocyte maturation**					
	CUST_11642	CNUS0000105941	Akt1;RAC serine/threonine-protein kinase	−6.92	0.022	0.005
	CUST_12915	CNUS0000107219	Protein RSKN-1	−2.48	0.018	0.005
	CUST_3675	CNUS0000097970	MAPK/ERK kinase 1	−4.15	0.0002	0
**Development/Reproduction**	**Dorso-ventral axis formation**					
	CUST_3675	CNUS0000097970	MAPK/ERK kinase 1	−4.15	0.0002	0
	CUST_3717	CNUS0000098012	Epidermal growth factor receptor	−4.52	0.004	0.005
	**Reproduction**					
	CUST_12936	CNUS0000107240	Prostatic spermine-binding protein (SBP)	−7.27	0.012	0.005
**Immune System**	**Natural killer cell mediated cytotoxicity**					
	CUST_3675	CNUS0000097970	MAPK/ERK kinase 1	−4.15	0.0002	0
**Endocytosis**	CUST_10516	CNUS0000104814	beta-adrenergic-receptor kinase	−2.05	0.0033	0
	CUST_3717	CNUS0000098012	Epidermal growth factor receptor	−4.52	0.004	0.005
	CUST_5301	CNUS0000099597	Heat shock 70 kDa protein cognate 4	−2.24	0.024	0.01
	CUST_8672	CNUS0000102970	Partitioning-defective 3 homolog (PAR-3)	−3.85	0.032	0.005
	CUST_9487	CNUS0000103785	Probable beta-arrestin	−2.33	0.040	0.007
**Amino Acid Metabolism**	CUST_4493	CNUS0000098788	Aspartate aminotransferase	−2.11	0.001	0.005
	CUST_6397	CNUS0000100693	Dopamine beta-hydroxylase	−3.02	0.037	0.012
	CUST_6212	CNUS0000100508	Aromatic-L-amino-acid decarboxylase	−4.70	0.029	0.01
**Carbohydrate Metabolism**	**Inositol phosphate metabolism**					
	CUST_10542	CNUS0000104840	Phosphatidylinositol-4-phosphate 3-kinase C2 domain-containing alpha (PI3K-C2α)	−2.43	0.025	0.008
	CUST_13086	CNUS0000107391	Phosphatidylinositol 4-kinase alpha (PI4Kα)	−2.03	0.027	0.01
	**Citrate cycle (TCA cycle),Pyruvate metabolism**					
	CUST_6418	CNUS0000100714	Pyruvate carboxylase	−2.61	0.002	0.005
	**Galactose metabolism, Amino sugar and nucleotide sugar metabolism**					
	CUST_7443	CNUS0000101740	Galactokinase 2(GALK2)	−3.99	0.019	0.008
**Glycan Biosynthesis and Metabolism**	**Glycosylphosphatidylinositol(GPI)-anchor biosynthesis**					
	CUST_7658	CNUS0000101956	Glycosylphosphatidylinositol anchor attachment protein 1(GPAA1)	−3.07	0.0016	0.005
	**N-Glycan biosynthesis**					
	CUST_9370	CNUS0000103668	Glycoprotein(Protein GLY-2)	−2.00	0.007	0.006

#### Signal transduction/Endocrine system/Development/Reproduction

Mitogen-activated protein kinases (MAPKs) are intracellular serine/threonine protein kinases. The study confirmed that the MAPK signal transduction pathway was present in most cells and transduction of extracellular signals to the cell and its nucleus played a crucial role in cellular biological responses, such as cell proliferation, differentiation, transformation, and apoptosis. MAPK signal transduction pathway was highly conserved evolutionarily both in prokaryotic and eukaryotic cells. The serine-threonine kinase Akt, also known as protein kinase B (PKB), is an important effector for phosphatidylinositol 3-kinase signaling initiated by numerous growth factors and hormones, and has also been implicated in many metabolic functions, such as protein and lipid synthesis, carbohydrate metabolism, and transcription [45]. The serine-threonine kinase Akt represents an important mediator of insulin action in worms and flies. In *C. elegans*, mutations in Akt result in alterations in development and aging, and Akt/PKB has been implicated as a critical regulator of growth and longevity [46], [47]. Other studies have reported that Akt1^−/−^ mice displayed a conspicuous growth impairment and demonstrated defects in both fetal and postnatal growth, which persisted into adulthood [48]. Akt1^−/−^ mice were smaller when compared to wild-type littermates. In addition, Akt1^−/−^ mouse embryo fibroblasts are more susceptible to apoptosis induced by the tumor necrosis factor receptor superfamily, UV irradiation, and serum withdrawal [49]. Nonetheless, much uncertainty remains concerning how this signaling pathway diverges to allow independent regulation of such disparate biological processes as metabolism, aging, and growth. In schistosomes from water buffalo, the Akt gene was significantly down-regulated (CUST_11642, FC −6.92), suggesting that Akt may be involved in growth retardation via MAPK signal transduction or the endocrine system.

The RSKN-1 protein is a downstream effector of MPK-1/ERK and is critical for dedifferentiation. Rskn-1 RNAi suppressed spermatocyte dedifferentiation and instead induced meiotic division in the *C. elegans* germline. These regulators are broadly conserved, suggesting that similar molecular circuitry may control cellular dedifferentiation in other organisms as well [50]. The Rskn-1 gene was underexpressed in schistosomes from water buffalo (CUST_12915, FC −2.48) and might also be associated with cellular dedifferentiation; however, this hypothesis requires further investigations.

KIN-18 encodes a previously uncharacterized protein in *C. elegans* and its catalytic domain shares over 60% identities with TAO kinase 1 (TAO1) and TAO2, which have been recently characterized with a MAPK/ERK kinase role during stress response. Berman et al. [51] reported that expression of constitutively active forms of TAO1/KIN-18 affect the physiology of intact worms and demonstrated that KIN-18 was a 120-kDa protein and its promoter was active in the pharynx and intestine of *C. elegans*. These worms grow more slowly, lay fewer eggs, and phenotypes could result from reduced feeding. Other research has suggested a role for MEK-1 in stress responses, with a focus in the pharynx and/or intestine [52]. The KIN-18 (CUST_9047, −15.09) and MEK-1 (CUST_3675, −4.15) genes were significantly downregulated in schistosomes from water buffalo, which may be attributed to the morphological defection in worms from water buffalo compared to those from goats, as reduced nutritional intake can retard worm growth.

EGFR belongs to the ErbB family of receptor tyrosine kinases, which possess protein tyrosine kinase activity and are found only in metazoans [53]. The downstream signaling proteins initiate several signal transduction cascades, principally the MAPK, Akt, and mitogen-activated protein kinase 8 isoform beta 2 pathways, leading to DNA synthesis and cell proliferation. EGFR plays an important role in development and cell differentiation, and EGFR homologues have been identified in a broad range of vertebrate and invertebrate organisms [54]. Here, we found that EGFR was differentially underexpressed in schistosomes from water buffalo (CUST_3717, −4.52). EGFR can act as a target regulated by host factors, resulting in different worm compatibility within different hosts, thereby supporting the hypothesis that host EGF can regulate *S. mansoni* development [55].

Hsp70 proteins are ubiquitously expressed and exist in virtually all living organisms. The Hsp70 proteins are an important part of the cellular machinery for protein folding and help to protect cells from thermal or oxidative stress [56]. In addition to improving overall protein integrity, Hsp70 directly inhibits apoptosis by blocking the recruitment of procaspase-9 to the Apaf-1/dATP/cytochrome c apoptosome complex [57]. Given that water buffalo is the less suitable host, the Hsp70 gene of schistosomes from water buffalo was down-regulated (CUST_5301 FC,-2.24), therefore Hsp70 is predicted to promote parasitic apoptosis in vivo [58].

Dsh is a family of proteins involved in canonical and non-canonical integration 1/wingless (Wnt) signaling pathways. Dsh is a cytoplasmic phosphoprotein that acts directly downstream of frizzled receptors [59] and plays important roles in both the embryo and the adult, ranging from cellular differentiation and cell polarity to social behavior [60]. However, the role of Dsh downregulation in schistosomes from water buffalo (CUST_5485, −4.48) has not yet been investigated.

Prostatic spermine-binding protein (SBP) binds to forkhead protein A1 (FoxA1) and FoxA1 is critical for the androgenic regulation of prostate-specific promoters. Androgen plays a key role in the normal prostate development and physiology [61]. Female worms require stimulation from male worms to achieve and maintain a mature reproductive state. The SBP gene was significantly underexpressed in schistosomes from water buffalo (CUST_12936,−7.27), which was predicted to have a role in reproduction processes by influencing interactions among male and female worms, resulting in parasitic retardation and less egg deposition in the liver [28].

#### Endocytosis/Amino acid/Carbohydrate metabolism/Glycan biosynthesis, and metabolism

Endocytosis is a process by which cells absorb biological macromolecules, including hormones, growth factors, lymphokines, and some nutrients, by engulfing them and is used by all cells of the body because most important substances are large polar molecules that cannot pass through the hydrophobic plasma or cell membrane [62]. Normal parasitic growth must be accompanied by basic metabolism and since schistosome survival and development in water buffalo was hindered, genes associated with endocytosis of nutrients, amino acid metabolism, carbohydrate metabolism, and glycan biosynthesis/metabolism-associated genes were accordingly downregulated.

A comparison of schistosomes from the less susceptible host (water buffalo) with those from the more susceptible host (goats) at the phenotype and gene expression levels. Except the length and width difference, SEM and TEM showed that the ultrastructure of worms from different hosts were very different. A total of 485 differentially expressed genes were identified and their gene expression patterns in schistosomes from water buffalo and goat hosts were identified here. Our results revealed that, compared with schistosomes from goats, genes involved in lipid metabolism, genetic information processing, folding, sorting and degradation were upregulated in schistosomes from water buffalo, whereas other genes associated with signal transduction, endocrine function, development, reproduction, endocytosis, amino acid/carbohydrate metabolism, immune function, etc. were downregulated. These events were deduced to be key differences for the survival and development of schistosomes in different compatible natural host environments.

The microarray analysis of gene expression differences in schistosomes derived from water buffalo and goat provided a useful platform to discover the differences in host compatibilities, and furthered our understanding of the interplay between natural hosts and parasites, and identified several schistosome target genes associated with susceptibility for the screening of vaccine candidates.

## Supporting Information

Table S1 **The common up-regulated genes in schistosomes from water buffalo compared with those from yellow cattle and goat.**
(DOC)

Table S2 **The common down-regulated genes in schistosomes from water buffalo compared with those from yellow cattle and goat.**
(DOC)
