# Supplementary material for: Microarray Analysis of Gene Expression Profiles of Schistosoma japonicum Derived from Less-Susceptible Host Water Buffalo and Susceptible Host Goat
Source: PLoS One. 2013 Aug 5;8(8):e70367. doi: 10.1371/journal.pone.0070367 (PMC3734127; doi:10.1371/journal.pone.0070367)
Supplement: Table S1 — The common up-regulated genes in schistosomes from water buffalo compared with those from yellow cattle and goat. (DOC) [file pone.0070367.s001.doc]

| ***Probe name*** | ***Acession number*** | ***Gene description*** | ***Protein homology*** | ***B_vs_C*** | | ***B_vs_G*** | |
| --- | --- | --- | --- | --- | --- | --- | --- |
|  |  |  |  | ***p* value** | **FC** | ***p* value** | **FC** |
| CUST_4112 | CNUS0000098407 | cyclin-dependent kinase 6 [EC:2.7.11.22] | cyclin-dependent kinase 6 | 0.029 | 18.52 | 0.047 | 6.34 |
| CUST_12988 | CNUS0000107293 | Putative uncharacterized protein C14orf165 | uncharacterized protein c14orf165-like | 0.007 | 8.86 | 0.048 | 12.56 |
| CUST_10057 | CNUS0000104355 | Protocadherin gamma B2 precursor (PCDH-gamma-B2) | protocadherin alpha subfamily 2 | 0.036 | 7.95 | 0.035 | 8.33 |
| CUST_10350 | CNUS0000104648 | Asparagine-rich protein (Ag319) (ARP) (Fragment) | ---NA--- | 0.003 | 6.11 | 0.010 | 6.88 |
| CUST_1782 | CNUS0000096077 | Eukaryotic translation initiation factor 3 subunit 12 (eIF-3 p25) (eIF3k) | eukaryotic translation initiation factor 3 subunit k | 0.009 | 5.33 | 0.020 | 3.88 |
| CUST_9981 | CNUS0000104279 | Serine/threonine-protein phosphatase 2A 56 kDa regulatory subunit alpha isoform (PP2A, B subunit, B' alpha isoform) | protein phosphatase regulatory subunit b alpha isoform | 0.000 | 4.54 | 0.009 | 2.94 |
| CUST_10723 | CNUS0000105021 | expressed protein | sjchgc03548 protein | 0.036 | 4.21 | 0.034 | 4.73 |
| CUST_2890 | CNUS0000097185 | Rrp4 [EC:3.1.13.-]; ko:K03679 RNA-binding protein Rrp4 and related proteins | exosome complex exonuclease | 0.049 | 3.96 | 0.020 | 6.13 |
| CUST_12961 | CNUS0000107266 | expressed protein | ---NA--- | 0.007 | 3.81 | 0.015 | 4.41 |
| CUST_488 | FN317168 | isolate Anhui full length mRNA clone | sjchgc08923 protein | 0.015 | 3.52 | 0.022 | 3.69 |
| CUST_1819 | CNUS0000096114 | hypothetical protein, mRNA | ---NA--- | 0.017 | 3.39 | 0.007 | 2.59 |
| CUST_4101 | CNUS0000098396 | Iroquois-class homeodomain protein IRX-3 (Iroquois homeobox protein 3) (Homeodomain protein IRXB1) | iroquois homeobox family transcription factor | 0.031 | 2.66 | 0.006 | 4.88 |
| CUST_6737 | CNUS0000101033 | hypothetical protein, mRNA | hypothetical protein | 0.039 | 2.58 | 0.012 | 3.62 |
| CUST_5733 | CNUS0000100029 | Elongation of very long chain fatty acids protein 1 | elongation of very long chain fatty acids (fen1 sur4 yeast)-like 1 | 0.039 | 2.57 | 0.039 | 2.38 |
| CUST_1079 | AY809022 | SJCHGC02234 protein mRNA, partial cds | sjchgc02234 protein | 0.031 | 2.56 | 0.022 | 3.14 |
| CUST_8181 | CNUS0000102479 | hypothetical protein, mRNA | hypothetical protein [] | 0.039 | 2.42 | 0.014 | 2.95 |
| CUST_694 | FN318567 | isolate Anhui full length mRNA clone | hypotheticial protein | 0.029 | 2.40 | 0.005 | 3.94 |
| CUST_11457 | CNUS0000105756 | Histone H4 | histone h4-like | 0.006 | 2.36 | 0.047 | 2.45 |
| CUST_11472 | CNUS0000105771 | hypothetical protein, mRNA | hypothetical protein [Schistosoma mansoni] | 0.002 | 2.34 | 0.009 | 2.77 |
| CUST_2605 | CNUS0000096900 | Histone H4 | histone h4-like | 0.009 | 2.27 | 0.038 | 2.20 |
| CUST_1041 | FN326681 | isolate Anhui full length mRNA clone | hypotheticial protein | 0.022 | 2.25 | 0.018 | 2.80 |
| CUST_2965 | CNUS0000097260 | LIM/homeobox protein Lhx1 (LIM homeobox protein 1) | lim homeobox transcription factor alpha | 0.024 | 2.23 | 0.001 | 3.14 |
| CUST_88 | f4-c06-t7p | ---NA--- | ---NA--- | 0.015 | 2.20 | 0.014 | 3.68 |
| CUST_738 | FN318954 | isolate Anhui full length mRNA clone | hypotheticial protein | 0.034 | 2.20 | 0.014 | 2.58 |
| CUST_8688 | CNUS0000102986 | expressed protein | sjchgc08962 protein | 0.028 | 2.19 | 0.010 | 3.54 |
| CUST_1940 | CNUS0000096235 | MGC81224; hypothetical protein MGC81224 [EC:3.6.3.14] | v-type proton atpase subunit e 1 | 0.040 | 2.19 | 0.004 | 2.53 |
| CUST_4513 | CNUS0000098808 | hypothetical protein, mRNA | ---NA--- | 0.015 | 2.18 | 0.015 | 2.51 |
| CUST_11483 | CNUS0000105782 | Ecotropic virus integration site 1 protein (EVI-1) | mds1 and evi1 complex locus protein evi1 | 0.012 | 2.12 | 0.010 | 2.11 |
| CUST_739 | FN318955 | isolate Anhui full length mRNA clone | hypotheticial protein | 0.033 | 2.12 | 0.014 | 2.85 |
| CUST_8420 | CNUS0000102718 | hypothetical protein, mRNA | pumilio [Schistosoma mansoni] | 0.040 | 2.11 | 0.040 | 2.03 |
| CUST_13750 | chgc_new_contig1468 | ---NA--- | ---NA--- | 0.002 | 2.10 | 0.011 | 3.10 |
| CUST_6370 | CNUS0000100666 | UPF0195 protein CG7949 | zgc:92345 protein | 0.050 | 2.09 | 0.040 | 2.28 |
| CUST_7137 | CNUS0000101434 | 39S ribosomal protein L35, mitochondrial precursor (L35mt) | 39s ribosomal protein mitochondrial | 0.010 | 2.08 | 0.027 | 2.04 |
| CUST_49 | chgc_new_contig1630 | ---NA--- | ---NA--- | 0.024 | 2.07 | 0.013 | 2.28 |
| CUST_12650 | CNUS0000106952 | Conserved hypothetical protein, expressed protein | lyr motif-containing protein 1 | 0.022 | 2.07 | 0.016 | 2.44 |
| CUST_2092 | CNUS0000096387 | Regulating synaptic membrane exocytosis protein 2 (Rab3-interacting molecule 2) (RIM 2) (Rab3-interacting protein 2) | rab3 interacting molecule -related | 0.018 | 2.07 | 0.005 | 2.82 |
| CUST_13757 | f06-d04 | ---NA--- | ---NA--- | 0.016 | 2.07 | 0.007 | 5.86 |
| CUST_5523 | CNUS0000099819 | Putative eukaryotic translation initiation factor 3 subunit (eIF-3) | eukaryotic translation initiation factor 3 subunit (eif-3) | 0.037 | 2.03 | 0.010 | 2.56 |
| CUST_7791 | CNUS0000102089 | LOC476638; similar to nucleoside diphosphate kinase type 6; ko:K00940 | nucleoside diphosphate kinase 6 | 0.027 | 2.02 | 0.033 | 2.21 |
| CUST_12289 | CNUS000010658 | hypothetical protein | ---NA--- | 0.035 | 2.01 | 0.048 | 3.68 |

The list including the probe name, gene accession, FC (the fold change) , regulation and the protein homology(the result of blastx). In the gene accession line, the name such as CNUS0000098407 is from LSBI (<http://lifecenter.sgst.cn/schistosoma/cn/genomeProject.do>), the name such as FN317168 is from EMBL (<http://www.ebi.ac.uk/embl/>), the name such as AY809022 is from GeneBank (<http://www.ncbi.nlm.nih.gov/genbank/>), the name such as chgc_new_contig1468 is from CHGC new contigs (<http://www.chgc.sh.cn/japonicum/Resources.html> ), the name such as f06-d04 is from our lab data(unpublished).
