# Supplementary material for: Microarray Analysis of Gene Expression Profiles of Schistosoma japonicum Derived from Less-Susceptible Host Water Buffalo and Susceptible Host Goat
Source: PLoS One. 2013 Aug 5;8(8):e70367. doi: 10.1371/journal.pone.0070367 (PMC3734127; doi:10.1371/journal.pone.0070367)
Supplement: Table S2 — The common down-regulated genes in schistosomes from water buffalo compared with those from yellow cattle and goat. (DOC) [file pone.0070367.s002.doc]

| ***Probe name*** | ***Acession number*** | ***Gene description*** | ***Protein homology*** | ***B_vs_C*** | | ***B_vs_G*** | |
| --- | --- | --- | --- | --- | --- | --- | --- |
|  |  |  |  | ***p* value** | **FC** | ***p* value** | **FC** |
| CUST_10799 | CNUS0000105097 | Conserved hypothetical protein, expressed protein | ---NA--- | 0.024 | 2.92 | 0.024 | 3.07 |
| CUST_11761 | CNUS0000106060 | hypothetical protein; arginine-tRNA-protein transferase | homeobox protein distal-less dlx | 0.043 | 2.22 | 0.010 | 5.83 |
| CUST_12613 | CNUS0000106914 | hypothetical protein | sjchgc02128 protein | 0.022 | 2.92 | 0.005 | 6.14 |
| CUST_4819 | CNUS0000099115 | solute carrier family 6 (neurotransmitter transporter, betaine/GABA), member 12; | taurine transporter | 0.006 | 2.07 | 0.007 | 2.14 |
| CUST_8162 | CNUS0000102460 | Krt9; keratin 9; ko:K07604 type I | ---NA--- | 0.014 | 2.73 | 0.018 | 2.55 |
| CUST_8465 | CNUS0000102763 | expressed protein | cell polarity protein leucine-rich repeat protein scribble complex protein | 0.001 | 2.70 | 0.000 | 7.44 |
